# Poor Host Status of Australian Finger Lime, *Citrus australasica*, to *Ceratitis capitata*, *Zeugodacus cucurbitae,* and *Bactrocera dorsalis* (Diptera: Tephritidae) in Hawai’i

**DOI:** 10.3390/insects13020177

**Published:** 2022-02-08

**Authors:** Peter A. Follett, Glenn Asmus, Lindsey J. Hamilton

**Affiliations:** USDA-ARS, Daniel K. Inouye U.S. Pacific Basin Agricultural Research Center, 64 Nowelo Street, Hilo, HI 96720, USA; glenn.asmus@usda.gov (G.A.); lindsey.hamilton@usda.gov (L.J.H.)

**Keywords:** citrus, nonhost, tephritidae, host status, quarantine pest, phytosanitary, Mediterranean fruit fly, melon fly, oriental fruit fly

## Abstract

**Simple Summary:**

Tephritid fruit flies are major economic pests for fruit production and impede international trade. Different host fruits are known to vary in their suitability for fruit flies to complete their life cycle. International regulatory standards that define the legal host status for tephritid fruit flies categorize fruits as a natural host, a conditional host, or a nonhost. For those fruits that are natural or conditional hosts, infestation rate can vary as a spectrum ranging from highly attractive fruits supporting large numbers of fruit flies to very poor hosts supporting low numbers. Finger lime, *Citrus australasica*, is a new crop in Hawai’i, and no information existed on its susceptibility to Hawai’i’s tephritid fruit fly pests. Host status testing was conducted using no-choice cage tests and field collections. *Ceratitis capitata* and *Zeugodacus cucurbitae* readily oviposited into finger limes, but individuals rarely developed to the pupal or adult stage. Finger lime was not infested by *Bactrocera dorsalis* and is probably a nonhost. For export, a postharvest treatment may still be required despite the very low infestation rate. Heat treatment, irradiation, and a systems approach are options to reduce the risk of exporting tephritid fruit flies in Hawai’i finger limes.

**Abstract:**

We examined the host status of the Australian finger lime, *Citrus australiasica* F. Muell. (Rutaceae), to Hawai’i’s tephritid fruit fly pests using laboratory and field studies. In high-density (500 flies, 1:1 males and females) no-choice cage exposures (25 × 25 × 25-cm cage size), both undamaged and punctured finger limes were infested by *Ceratitis capitata* and *Zeugodacus* *cucurbitae* at a low rate compared to papaya controls, whereas *Bactrocera dorsalis* did not infest undamaged fruit, suggesting finger lime is a nonhost. In low-density (50 females) no-choice cage exposures, *C. capitata* and *Z. cucurbitae* readily oviposited in undamaged fruit but individuals rarely developed to the pupal or adult stage. For *C. capitata*, 274 finger limes exposed to 2000 gravid females, which laid an estimated 14,384 eggs, produced two pupae and no adults. For *Z. cucurbitae*, 299 fruit exposed to 2000 gravid females, which laid an estimated 4484 eggs, produced four pupae and one adult. Field sampling of undamaged fruit from the tree and off the ground from commercial farms produced five *C. capitata* pupa and one adult from 1119 fruit, for an infestation rate of 0.05 flies per kilogram of fruit; field collections found no natural *Z. cucurbitae* or *B. dorsalis* infestation, but the number of fruit available was too low to demonstrate nonhost status with a high degree of confidence.

## 1. Introduction

The Australian finger lime, *Citrus australasica* F. Muell. (Rutaceae), is native to the subtropical rainforests in the mountain ranges bordering southeast Queensland and northwest New South Wales [1,2]. Finger lime is a thorny understory tree that naturally grows as a shrub or tree up to 6 m in height on a range of soil types. The finger-shaped fruit can vary in size (up to 10 cm in length), shape, color and seediness, and the pulp is unique with compressed small round juice vesicles that have a caviar-like appearance. Skin color can range from yellowish green, to crimson, purple, or black, and pulp color can vary from yellowish green to pale pink to coral to scarlet. Finger lime is thought to have the widest range of color variation within any *Citrus* species and has been popularized in Australia as a gourmet bushfood [1].

Export of *Citrus* spp. fruits may require risk mitigation if grown in areas with established tephritid fruit fly populations capable of infesting the fruits [3,4]. In Hawai’i, the main fruit flies of concern in *Citrus* spp. are Mediterranean fruit fly, *Ceratitis capitata* (Wiedemann); melon fly, *Zeugodacus cucurbitae* (Cocquillett); and oriental fruit fly, *Bactrocera dorsalis* (Hendel) (Diptera: Tephritidae). Currently, *Citrus* spp. in Hawai’i may be exported to the continental United States using heat or irradiation quarantine treatments to control fruit flies [5]. Different *Citrus* species and varieties can vary widely in their susceptibility to a particular tephritid fruit fly species [6]. Because finger lime is a newly cultivated crop in Hawai’i and is a new association with Hawai’i’s polyphagous fruit flies, an evaluation was required to determine the fruit’s susceptibility to infestation. 

Hosts for fruit flies are plants (fruits or vegetables) on which flies are able to lay eggs and complete their whole life cycle through to adult emergence [7]. To complete its entire life cycle on a fruit or vegetable, a fruit fly must first find the plant and accept it for egg laying. The eggs must hatch, and the resultant larvae must survive and develop on the tissues of the fruit or vegetable; reach maturity; and give rise to healthy, sexually competent adults [8,9]. Any plants that do not allow flies to produce viable adult offspring are, by definition, a nonhost. Different host fruits are known to vary in their suitability to fruit flies to complete their life cycle [6]. Currently, international regulatory standards that define the potential legal host status for tephritid fruit flies categorize fruits as a natural host, a conditional host, or a nonhost [10]. For those fruits that are natural or conditional hosts, infestation rate can vary as a spectrum ranging from highly attractive fruits supporting large numbers of fruit flies to very poor hosts supporting low numbers. Several methods have been proposed for the determination of host status of fruit; to fruit flies which rely primarily either on laboratory no-choice cage testing as proposed by Cowley et al. [11]; on natural and semi-natural field testing as per the International Standards for Phytosanitary Measures (ISPM) No. 37 [10], or on both [6].

Previous studies with finger limes and fruit flies are limited. In Australia, Jessup [12] showed that finger lime is a nonhost for Queensland fruit fly, *Bactrocera tryoni*, suggesting finger lime may be a nonhost for other polyphagous tephritid fruit flies and therefore may not require risk mitigation for export. Finger lime is grown year-round in Hawai’i on a small scale but has the potential for expansion if fruit could be exported to markets in the continental United States and elsewhere. In the present study, we tested the host status of finger lime to *C. capitata*, *Z. cucurbitae,* and *B. dorsalis* by using no-choice cage experiments and field collections. Our hypothesis was that finger lime might be a nonhost for these three fruit fly species.

## 2. Materials and Methods

### 2.1. Experimental Insects and Fruit

*Ceratitis capitata*, *Zeugodacus cucurbitae*, and *Bactrocera dorsalis* were obtained from colonies maintained at the USDA-ARS, United States Daniel K. Inouye Pacific Basin Agricultural Research Center in Hilo, Hawai’i on standard diets [13]. These fruit fly colonies have been maintained for 20–30 years (~200–400 generations), with periodic infusion of wild flies. Fruit flies used in our tests were maintained in an insectary at 24–27 °C, 65–70% RH, and a photoperiod of 12:12 (L:D) hours. Approximately 500 adult flies were allowed to emerge inside cubical screen cages (25 × 25 × 25 cm). Fruit flies were supplied with a 3:1 mixture of sucrose and USB enzymatic yeast hydrolysate (United States Biochemical, Cleveland, OH, USA) as a food source, and water ad libitum. Adult flies were 12–14 days old at the time of testing, and females were reproductively mature and actively laying eggs.

**Figure 1:**
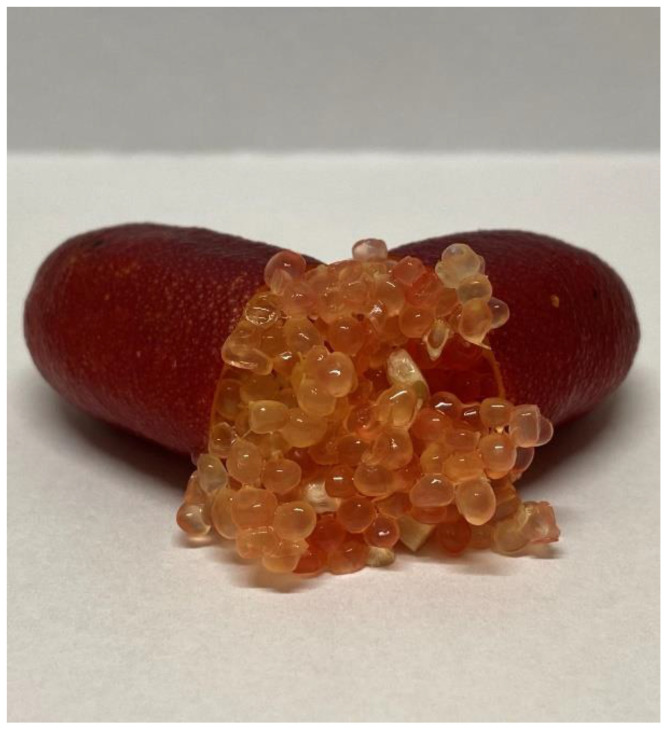
‘Red Champion’ finger lime (photo credit: Oliver Cohen).

‘Red Champion’ finger limes (Figure 1) with stems attached were collected from the Aloha Honey Farm (elevation, 89 m; 22.2119° N, 159.4122° W) near Kilauea on the island of Kauai, Hawai’i. This 3-ha farm contains approximately 100 finger lime trees in a block planted near other tropical fruit trees. All tests used harvest mature finger limes freshly harvested from trees. Finger limes do not ripen after harvest. Fruits were harvested and shipped the same day from Kauai to the laboratory at USDA-ARS in Hilo, Hawai’i two days before testing.

### 2.2. High Density Cage Test—Adult Emergence

Approximately 100 g of fruit (6–9 individual fruit) was placed in a single layer on the floor of 25 × 25 × 25-cm screen cages with 500 (high-density) adult male and female (1:1 sex ratio) fruit flies for 24 h in a no-choice test. The high-density test was conducted indoors under constant light (24:0 h) at 25 °C. The 24-h exposure period and constant light were used to maximize the chance of oviposition and infestation. All fruit in a cage were either undamaged (without damage or blemishes) or punctured individually with six punctures (two rows of three) using a No. 5 insect pin. Puncturing is a way to intentionally damage fruit and facilitate female oviposition [6]. No-choice cage exposures with high insect density were replicated five times for undamaged and punctured fruit. Observations were made on the general behavior of each fruit fly species toward the finger limes (landing and walking on fruit, and attempted oviposition), but specific behaviors were not quantified. Ripe ‘Rainbow’ papaya, which is a preferred host for *C. capitata*, *Z. curcurbitae,* and *B. dorsalis*, was exposed similarly as a control to demonstrate that gravid females were behaviorally and physiologically ready to lay eggs [14]. Papayas were exposed as a single fruit per cage and replicated two times for each fruit fly species. After 24 h exposure to fruit flies, each 100 g group of finger lime fruit and papaya was placed in a 3.8-L plastic bucket with a screened lid and held at 20–25 °C. Approximately 300 g of sand was added to the bucket as a pupation medium. At two weeks and three weeks post infestation, the sand was sieved and inspected for pupae, and pupae were transferred to 120-mL plastic cups for adult emergence. At three weeks, each fruit was dissected and inspected for remaining larvae. 

### 2.3. Low Density Cage Test—Oviposition

**Figure 2:**
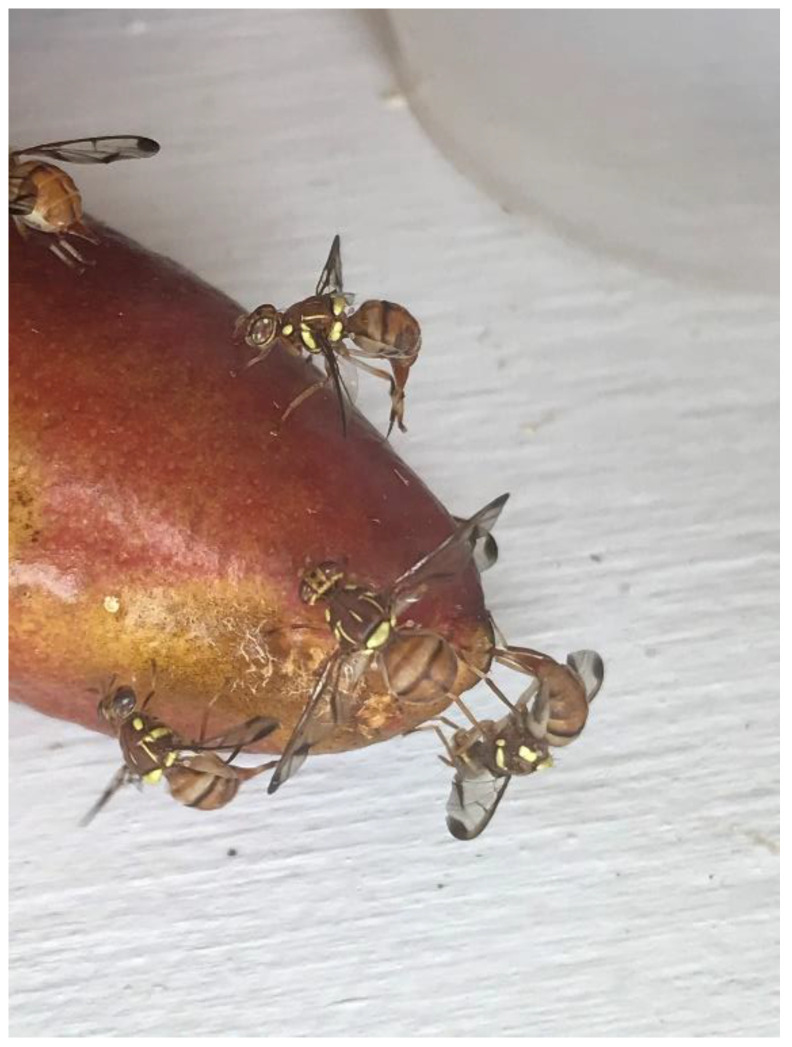
Melon flies (*Zeugodacus cucurbitae*) ovipositing on a finger lime in the no-choice cage test with a low fruit fly density.

Both *C. capitata* and *Z. cucurbitae* showed significant oviposition behavior in punctured (damaged) and undamaged fruit during the high-density cage test (Figure 2), and further testing was conducted with these species to quantify egg-laying in fruit with a lower density of flies. *Bactrocera dorsalis* was omitted from further testing because it showed limited host acceptance and oviposition behavior in finger limes in the high-density cage test and because no adults emerged from undamaged fruit. For *C. capitata* and *Z. cucurbitae*, approximately 100 g of fruit (6–9 individual fruit) was placed in a single layer on the floor of 25 × 25 × 25-cm screen cages with 50 (low-density) gravid females for 24 h in a no-choice test following Cowley et al. [10]. The no-choice cage test with a low fruit fly density was conducted under semi-natural conditions outdoors under a roof with natural light (12:12 h) and temperature (16.5–25 °C). The 24-h exposure period was used to maximize the chance of oviposition and infestation. In the low-density test, all fruit were premium quality and without damage or blemishes. The no-choice cage exposures with a low fruit fly density for oviposition were replicated seven times for each species. After the 24 h exposure to fruit flies, fruit were dissected and eggs were counted. Examination for eggs was destructive to the fruit, so collecting data on hatch and development was not possible. 

### 2.4. Low Density Cage Test—Adult Emergence

The low-density no-choice test described above was repeated but with a focus on fruit fly development in fruit, as evidenced by pupation and adult emergence. For each species, 100 g of fruit was placed in 25 × 25 × 25 cm screen cages with 50 gravid female fruit flies for 24 h in a no-choice test following Cowley et al [11]. The test was conducted outdoors under a roof with natural light (12:12 h) and temperature (16.5–25 °C) conditions. Fruit flies in cages were provided supplementary protein and sugar, in addition to fruit. All fruits were premium quality and without damage or blemishes. No-choice cage exposures with a low fruit fly density to examine infestation and development to the adult stage were replicated 10 times for each species on four separate dates for a total of 40 replicates and 250–300 fruit. On each date, three additional cages containing one ripe papaya each were exposed similarly as controls to demonstrate oviposition competence [13]. After 24 h exposure to fruit flies in the low-density cage tests, the fruit from each cage was placed in a 3.8-L plastic bucket with a screened lid and held at 20–25 °C. Approximately 300 g of sand was added to the bucket as a pupation medium. At two weeks and three weeks post infestation, the sand was sieved and inspected for pupae, and pupae were transferred to 120-mL plastic cups for adult emergence. All pupae from the finger limes were weighed, and 10 individual pupae were randomly selected from each papaya and weighed. At three weeks, finger lime and papaya fruits were dissected and inspected for any remaining larvae. 

### 2.5. Field Collection of Fruit

Finger lime fruit were sampled from trees during harvest and observed for natural infestation following ISPM 37 [10]. Finger limes (‘Red Champion’) from the tree and off the ground beneath the tree were collected randomly from multiple trees on seven dates at two- to three-week intervals from May to October 2021 at Aloha Honey Farm and shipped to the USDA-ARS laboratory in Hilo, Hawaii. Fruits were held in groups of 10 fruit in containers in the laboratory for fruit fly emergence as described above. Finger limes (both green and red fruit types) were also collected from several trees on the Love Family Farm (elevation, 335 m; 19.5007 N, −155.9211 W) in Captain Cook on the island of Hawai’i and held for any fruit fly emergence. Any pupae that developed from fruit were weighed. This farm has a small number of finger lime trees interspersed with a wide variety of different tropical fruits. McPhail traps containing protein bait lures (torula yeast in water) were suspended in two locations on both farms to monitor the *C. capitata*, *Z. cucurbitae,* and *B. dorsalis* populations and demonstrated their presence on the two commercial farms during the time of field sampling.

### 2.6. Statistical Analysis

For the high-density cage test, separate statistical analyses were performed for each fruit fly species for performance on punctured and undamaged finger limes and papayas. The treatments were fruit type (finger limes or papaya) and puncture status (punctured or undamaged). Analysis of variance (ANOVA) was performed on response data for pupal and adult recovery per kilogram of fruit [14]. A log (x + 1) transformation was applied to pupal and adult recovery counts to improve normality and equality of variances. For significant effects, means separations were performed using a Tukey’s test at α = 0.05. For the low-density oviposition test, a *t*-test was performed on the number of eggs per fruit and number of eggs per kg fruit for *C. capitata* and *Z. cucurbitae* after log (x + 1) transformation. For the low-density cage test, a nonparametric Wilcoxon rank sum test was performed on pupal and adult recovery data from finger limes and papaya for *C. capitata* and *Z. cucurbitae*, and significance was reported as a two-sample chi-square (χ^2^) approximation [15]. Untransformed data are presented in the summary tables. 

## 3. Results

### 3.1. High Density Cage Test—Adult Emergence

In the high-density test, development of pupae and emergence of adults was low for both undamaged and punctured finger limes for all three fruit fly species compared to the preferred host papaya (Table 1). The effect of fruit status (punctured finger limes, undamaged finger limes, and papaya controls) was highly significant for development of pupae per kg fruit in *C. capitata* (F_2,11_ = 9.1, *p* = 0.007), *Z. cucurbitae* (F_2,11_ = 6.9, *p* = 0.01), and *B. dorsalis* (F_2,11_ = 27.5, *p* < 0.001). The effect of fruit type (undamaged finger limes, punctured finger limes, papaya controls) was also highly significant for adult emergence per kg fruit in *C. capitata* (F_2,11_ = 14.0, *p* = 0.002) and *B. dorsalis* (F_2,11_ = 21.6, *p* < 0.001) but less significant in *Z. cucurbitae* (F_2,11_ = 2.0, *p* = 0.20) due to poor emergence in papaya. The large number of *Z. cucurbitae* larvae developing in the papaya apparently overwhelmed the resource, resulting in small-sized pupae and low adult emergence. For *C. capitata* and *Z. cucurbitae*, the number of individuals developing to pupae and emerging as adults was not significantly different between undamaged and punctured finger limes (Table 1). No *B. dorsalis* pupae developed in undamaged finger limes, and therefore finger lime was declared a nonhost for *B. dorsalis,* and this fruit fly was omitted from further study. In terms of behavior, both *C. capitata* and *Z. cucurbitae* showed strong interest in finger limes, with many flies landing and walking on fruit and attempting to oviposit, whereas *B. dorsalis* showed minimal interest, with few flies landing on fruit despite the high density of flies in the cages.

### 3.2. Low Density Cage Test—Oviposition

In the low-density oviposition test, *C. capitata* laid significantly more eggs overall than *Z. cucurbitae* (t_1,99_ = 8.3, *p* = 0.005) (Table 2). The mean number of eggs laid per infested fruit was not significantly different between the two fruit fly species (t_1,21_ = 0.02, *p* = 0.89), but *Z. cucurbitae* laid eggs in only five out of 50 fruits, whereas *C. capitata* laid eggs in 17 or of 50 fruits. Finger lime fruit weight ranged from 6.7 to 21.7 g (mean weight (+SE) = 13.5 (0.50) g), but fruit weight was not a significant factor in the number of eggs laid for *C. capitata* (t_1,16_ = 0.0002, *p* = 0.99) or *Z. cucurbitae* (t_1,4_ = 0.08, *p* = 0.80).

### 3.3. Low-Density Cage Test—Adult Emergence

Although *C. capitata* and *Z. cucurbitae* were shown to lay many eggs in finger limes (Table 2), the low-density adult emergence test suggested that egg hatch and development to pupae and adult emergence is very low (Table 3), thus demonstrating that finger lime is inherently a poor host. For *C. capitata*, 274 fruits exposed to 2000 gravid females (40 cages with 50 females each) produced only two pupae and no adults. For *Z. cucurbitae*, 299 fruits exposed to 2000 gravid females produced three pupae and only one adult fly emerged. The papaya fruit controls, meanwhile, produced 1000s of pupae and adult flies (Table 3). The difference in the mean number of pupae and adults produced in finger limes and papayas was highly significant for *C. capitata* (χ^2^ test, *p* < 0.001) and *Z. cucurbitae* (χ^2^ test, *p* < 0.001). *Ceratitis capitata* pupae reared on finger limes averaged 2.4 mg in weight, which was significantly less than pupae reared on papaya, which averaged 8.1 mg (F_1,99_ = 42.3, *p* < 0.001). *Zeugodacus cucurbitae* pupae reared on finger limes averaged 2.5 mg in weight, which was significantly less than pupae reared on papaya, which averaged 10.8 mg (F_1,104_ = 13.7, *p* < 0.001).

The information from Table 1 on oviposition and Table 2 on adult emergence in the low-density cage tests can be combined to estimate the survival rate from egg to pupa and egg to adult for *C. capitata* and *Z. cucurbitae*. In the oviposition test, *C. capitata* laid an average of 359.6 eggs per cage (2587 total eggs from 7 cages) and *Z. cucurbitae* laid an average of 112.1 eggs per cage (785 total eggs from 7 cages). If oviposition occurred at an equal rate in the adult emergence test with 40 replicate cages, *C. capitata* would have laid a total of 14,384 eggs (359.6 egg per cage × 40 cages) and *Z. cucurbitae* would have laid a total of 4484 eggs (112.1 eggs per cage × 40 cages). In this scenario, survivorship for *C. capitata* from egg to pupa and egg to adult would be 0.00014 (2/14.384) and 0.0 (0/14,384), and survivorship for *Z. cucurbitae* from egg to pupa and egg to adult would be 0.00067 (3/4484) and 0.00022 (1/4484), respectively. Poor survivorship is typically associated with poor growth [14], which was reflected in the average pupal weight in the surviving *C. capitata* and *Z. cucurbitae* reared from finger limes, which was 30% and 23%, respectively, of the weight of pupae reared from the papaya controls. Thus, finger limes showed a high degree of resistance to fruit fly survival and development.

### 3.4. Field Collection of Fruit

Field sampling of finger limes from the tree and off the ground at two commercial farms yielded only five pupae and one adult *C. capitata* and no *Z. cucurbitae* or *B. dorsalis* from a total of 1119 fruit (Table 4). McPhail trapping captured *Z. cucurbitae* and *B. dorsalis* on the commercial finger lime farm (Aloha Honey Farm) and only *B. dorsalis* on the mixed fruit farm (Love Family Farm).

## 4. Discussion

Determining the host status of a fruit for a particular fruit fly is an important part of pest risk analysis, and such determinations are required before developing export protocols for a new fruit in international trade. Because finger lime is a newly cultivated fruit outside Australia with a limited geographic distribution, historical evidence, pest interception records, and scientific literature are not available to allow a determination for most tephritid fruit fly species. Thus, laboratory and field studies were required to determine the host status of the Australian finger lime to the three economically important fruit flies in Hawai’i. In the present study, laboratory tests showed that finger lime is a very poor host for Mediterranean fruit fly (*Ceratitis capitata*) and melon fly (*Zeugodacus cucurbitae*), and a nonhost for oriental fruit fly (*Bactrocera dorsalis*). This result may have been partly due to the method we chose for exposing fruit to fruit flies. No-choice cage testing with fruit exposed to fruit flies in a confined space and high fly pressure is appropriate for demonstrating nonhost status but may suggest higher fruit susceptibility to fruit flies than occurs in nature [6]. The most reliable confirmation of natural host status comes from tree sampling of mature fruit during harvest and observation of natural infestation and development of viable adults in fruit. In our study, field sampling from commercial farms in Hawai’i demonstrated that finger lime can be naturally infested by *C. capitata* but at a very low rate. No *Z. cucurbitae* or *B. dorsalis* were found in field collected fruit, suggesting finger lime may be a natural nonhost for these fruit fly species. 

Follett et al. [6] proposed a Host Suitability Index (HSI) that divides host status into five categories based on the log infestation rate (number of flies per kilogram of fruit) ranging from very poor (<0.1), to poor (0.1–1.0), to moderately good (1.0–10.0), to good (10–100), to very good (>100). Infestation rates may be determined by cage infestation studies or field sampling. From the results in the low-density no-choice cage test for adult emergence (Table 3), finger limes would be characterized as a poor host for *C. capitata* based on pupation rate (0.51 pupae per kilogram of fruit) or a very poor host based on adult emergence (0.0 adults per kg of fruit). Finger limes would be characterized as a poor host for *Z. cucurbitae* based on both pupation rate (0.5 pupae per kilogram of fruit) and adult emergence (0.25 adults per kilogram of fruit). From field sampling (Table 4), only one *C. capitata* was reared from finger limes for an infestation rate of 0.05 adult flies per kilogram of fruit, underscoring its very poor host status. Although *Z. cucurbitae* and *B. dorsalis* meet the definition of a nonhost from field sampling (no adult flies emerging from fruit), the number of fruits sampled (total of 1119 fruit) was too low to show nonhost status with a high degree of confidence [12,16].

The mechanism for the high level of resistance in finger limes to the three fruit flies was not determined. Oviposition preference in *C. capitata* and other tephritid fruit flies in *Citrus* can be influenced by color, peel oil concentration, peel thickness and toughness, sugar content, and acidity [17,18,19]. Sugar content, acidity, and plant essential oils may also influence offspring performance [20]. Lemon is consistently the poorest larval host among commercial *Citrus* types for *C. capitata* [21,22,23] and *Z. cucurbitae* [4]. Our results with *C. capitata*, *Z. cucurbitae,* and *B. dorsalis*, and previous results with *B. tryoni* [12], suggest finger lime, like lemon, is generally a very poor host for tephritid fruit flies. 

Field control of tephritid fruit flies in finger lime using insecticides is probably not economical due to the very low rate of infestation and therefore minimal crop loss. For export, a postharvest treatment may still be required despite the very low infestation rate. Several postharvest quarantine treatments or approaches are available to control tephritid fruit flies in *Citrus* sp., thus allowing export from Hawai’i to the continental U.S., including heat treatment and irradiation [4,5]. High temperature forced air treatment is approved for control of *C. capitata*, *Z. cucurbitae,* and *B. dorsalis* in Hawai’i *Citrus* sp. and involves raising the fruit core temperature to a minimum pulp temperature >47.2 °C (117° F) in not less than 4 h. This treatment is organic. Multiple heat treatment facilities exist in Hawai’i for export of papayas to the continental U.S and Japan, which could be used for export of other crops such as finger lime. Irradiation at 150 Gy is an approved generic treatment for any tephritid fruit fly species [5,24] and could be used to export finger lime; if surface pests are found on *Citrus* sp. such as the spherical scale, *Nipaecoccus viridis*, or the tuckerellid mite, *Tuckerella ornanta*, a higher 400 Gy dose may be required [5]. Irradiation is not organic. Two irradiation facilities are available in Hawai’i for treatment of fresh fruit for export, and finger lime could be added to the list of approved crops for treatment [25]. Fruit quality information is not yet available for finger limes for heat treatment or irradiation. The development of a systems approach is an alternative to a stand-alone postharvest treatment and systems approaches are often developed around the fruit being a poor or rarely infested host [6,26]. A systems approach integrates multiple independent phytosanitary measures to cumulatively provide quarantine security [27]. In addition to poor host status, components of a systems approach might include pest survey, trapping and sampling, field treatment, sanitation, postharvest safeguards, and a restricted harvest and export period [28]. The availability of postharvest treatment options for exporting fruit will facilitate expansion of finger lime production in Hawai’i.

## Figures and Tables

**Table 1:** Host suitability of finger lime fruit to three tephritid fruit flies using no-choice laboratory cage exposures with 500 flies (~1:1, male:female) for 24 h.

Species Fruit Status	Replicates	Total No. Fruit	Total Fruit Weight ^a^	Total No. Pupae	Mean No. Pupae per kg Fruit ^b^	Total No. Adults	Mean No. Adults per kg Fruit
***Ceratitis capitata***							
Undamaged	5	35	488.7	1	2.1 (2.1) a	1	2.1 (2.1) a
Punctured	5	39	504.0	10	19.7 (12.7) a	2	3.9 (3.9) a
Papaya (control)	2	2	895.0	268	299.0 (76.5) b	226	251.8 (123.7) b
***Zeugodacus cucurbitae***							
Undamaged	5	40	506.7	24	47.7 (40.6) a	15	29.8 (23.1) a
Punctured	5	40	507.3	6	12.0 (12.0) a	5	9.9 (9.9) a
Papaya (control)	2	2	1482.2	1896	1270.4 (84.9) b	122	76.8 (52.7) a
***Bactrocera dorsalis***							
Undamaged	5	33	502.5	0	0.0a	--	--
Punctured	5	31	504.1	17	33.9 (16.9) b	7	14.1 (11.7) a
Papaya (control)	2	2	864.5	1324	1549.4 (141.3) c	811	957.5 (152.8) b

^a^ Finger limes weights are totals from five cages with approximately 100 g of fruit each; ^b^ means followed by different letters are significantly different by a Tukey’s HSD test at α = 0.05.

**Table 2:** Oviposition rate in finger lime fruit by *Ceratitis capitata* and *Zeugodacus cucurbitae* in no-choice cage exposures with 50 gravid flies for 24 h.

Species	No. Cages	Total No. Fruit	No. Infested Fruit	Total Fruit Weight (g)	Total No. Eggs	Mean No. Eggs per Fruit (+SE) ^a^	Mean No. Eggs per Infested Fruit (+SE) ^a^	Mean No. Eggs per kg Fruit (+SE) ^a^
*Ceratitis capitata*	7	50	17	673.6	2587 a	51.7 (13.4) a	152.2 (31.2) a	3965.0 (1128.9)
*Zeugodacus cucurbitae*	7	50	5	675.4	785 b	15.3 (6.1) b	141.8 (61.1) a	994.1 (493.9)

^a^ Means were calculated from replicate cage averages; means followed by different letters are significantly different by a Tukey’s HSD test at α = 0.05.

**Table 3:** Adult emergence from finger lime fruit by *Ceratitis capitata* and *Zeugodacus cucurbitae* in no-choice cage exposures with 50 gravid flies for 24 h.

Species	No. Cages (Replicates)	No. Cages with Infested Fruit	Total No. Fruit	Total Fruit Weight (g)	Total Pupae	Mean No. Pupae per kg Fruit ^a^	Total Adults	Mean No. Adults per kg Fruit ^a^
*Ceratitis capitata*								
Finger lime	40	2	274	4004.3	2	0.51 (0.29) *	0	0.0 *
Papaya	12	12	12	5741.4	5770	1010.2 (362.9)	3531	629.0 (252.2)
*Zeugodacus cucurbitae*								
Finger lime	40	2	299	4047.0	3	0.74 (0.47) *	1	0.25 (0.25) *
Papaya	12	12	12	4323.9	8399	1299.1 (959.2)	4245	692.7 (507.9)

^a^ Means were calculated from replicate cage averages; an asterisk (*) indicates a significant 1-way chi-square approximation from Wilcoxon rank sum test.

**Table 4:** Field infestation of finger lime fruit collected from off the tree and off the ground at two farms.

Farm	Replicates ^a^	No.	Total Fruit	*Ceratitis capitata*		*Zeugodacus cucurbitae*		*Bactrocera dorsalis*	
		Fruit	Weight (g)	Total No. Pupae	Total No. Adults	Total No. Pupae	Total No. Adults	Total No. Pupae	Total No. Adults
Aloha Honey									
Tree	50	447	5973.8	3	0	0	0	0	0
Ground	29	269	4109.5	2	1	0	0	0	0
Love Family									
Tree	9	76	692.1	0	0	0	0	0	0
Ground	34	327	3832.4	0	0	0	0	0	0

^a^ Each replicate included 8–10 fruit.

## Data Availability

Data can be obtained by contacting the corresponding author.
